# The Forgotten Test: Missed Opportunities for HIV Diagnosis and Survival Outcomes in Advanced HIV Disease

**DOI:** 10.3390/v18030356

**Published:** 2026-03-15

**Authors:** Arianna Narducci, Alessandra Vergori, Paola Borrelli, Irene Francesca Bottalico, Sergio Maria Ferrara, Chiara Grillo, Francesco Rosario Paolo Ieva, Mariacristina Poliseno, Annalisa Saracino, Antonio Cianciaruso, Riccardo Schiavoni, Laura Mezzogori, Antonio Di Biagio, Andrea Santoro, Giulia Carla Marchetti, Camilla Tincati, Sergio Lo Caputo

**Affiliations:** 1 Infectious Diseases Unit, A.O.U. Policlinico Foggia, University of Foggia, 71122 Foggia, Italy; arianna.narducci@gmail.com (A.N.); ibott4@gmail.com (I.F.B.); sferrara@ospedaliriunitifoggia.it (S.M.F.); chiaragrillo.infettivi@gmail.com (C.G.); francesco-rosario@hotmail.com (F.R.P.I.); sergio.locaputo@unifg.it (S.L.C.); 2 Laboratory of Biostatistics, Department of Medical, Oral and Biotechnological Sciences, University “G. D’Annunzio” Chieti-Pescara, 66013 Chieti, Italy; paola.borrelli@unich.it; 3 Clinic of Infectious Diseases, Department of Regenerative and Precision Medicine and Jonian Area (DiMePre-J), University of Bari, 70121 Bari, Italy; polisenomc@gmail.com (M.P.); annalisa.saracino@uniba.it (A.S.); antociancia@hotmail.it (A.C.); 4 Infectious Diseases Unit, AOM IRCCS, DiSSal University of Genova, 16132 Genova, Italy; riccardo.schiavoni@live.it (R.S.); lauramezzogori@yahoo.com (L.M.); antonio.dibiagio@hsanmartino.it (A.D.B.); 5 Infectious Diseases Unit, ASST Santi Paolo e Carlo, University of Milano, 20122 Milano, Italy; andrea.santoro1@unimi.it (A.S.); giulia.marchetti@unimi.it (G.C.M.); camilla.tincati@unimi.it (C.T.)

**Keywords:** advanced HIV diseases (AHD), HIV testing, AIDS presenters, missed opportunity

## Abstract

**Background:** Advanced HIV disease (AHD) remains highly prevalent and is associated with increased morbidity and mortality. Missed opportunities for early diagnosis continue to represent a major public health challenge. **Methods:** We conducted a multicenter retrospective cohort study including antiretroviral-naive people with HIV (PWH) presenting with AHD (CD4 < 200 cells/µL and/or AIDS) diagnosed between 1 January 2019 and 31 December 2024 in four Italian infectious diseases units. Demographic, clinical and viro-immunological data were collected at baseline and during follow up. Information on healthcare contacts, HIV-related symptoms, and prior HIV testing in the two years preceding diagnosis was obtained through structured interviews. **Results:** Among 658 newly diagnosed participants with HIV, 224 (34%) presented with AHD, of whom 54% presented with AIDS. Most participants (86.2%) had never undergone HIV testing before diagnosis. In the year preceding diagnosis 29.3% accessed healthcare services for symptoms compatible with HIV infection without being tested for HIV. At one year, 84.2% achieved virological suppression, with a median CD4 count of 260 cells/µL. Overall loss to follow-up was 27.2%. Five-year survival was significantly higher in non-AIDS presenters compared with AIDS presenters (100% vs. 85%, *p* = 0.005). **Conclusions:** Missed diagnostic opportunities remain frequent among PWH presenting with AHD, despite prior healthcare contacts. Wider implementation of indicator condition-guided HIV testing is urgently needed to reduce late diagnosis and improve long-term outcomes.

## 1. Introduction

The widespread availability of antiretroviral therapy (ART) has transformed HIV infection into a chronic condition, substantially reducing AIDS-related morbidity, mortality and transmission. Nevertheless, a considerable proportion of people with HIV (PWH) still present to care with advanced HIV disease (AHD), defined by a CD4 count below 200 cells/mmc and/or AIDS-defining conditions. These individuals remain at high risk of opportunistic infections, hospitalization, loss to follow up and premature mortality, even in the modern treatment era [1,2,3].

Advanced HIV infection has been described as a neglected condition, affecting more than 4 million people worldwide and accounting for over 600,000 deaths each year, many of which are potentially preventable through earlier diagnosis and timely linkage to care [2]. Despite universal access to HIV testing and treatment in many settings, late diagnosis continues to represent a major public health challenge.

In Europe, surveillance data from 2023 reported 112,883 new HIV diagnoses in 47 of the 53 countries in the European Region, with more than half (52,4%) classified as late presenters (CD4 cell count < 350 cells/µL) [4].

Similar patterns were observed in Italy in 2024, where late diagnosis remains particularly prevalent, with approximately 60% of new diagnoses occurring at CD4 count < 350 cells/µL, and over 40% presenting with AIDS-defining conditions [5]. Notably, the proportion of individuals presenting with severe immunosuppression has remained largely unchanged over the past decade, indicating persistent failures in timely HIV case detection and linkage to care [4,6,7,8]. AHD is associated with high short-term mortality and poor quality of life [9,10,11,12,13,14,15,16,17,18,19]. Clinical trials and observation studies report mortality rates ranging from 13% to over 20% within the first months after ART initiation, particularly among individuals with very low CD4 counts and those hospitalized with opportunistic infections, including tuberculosis, with the highest mortality occurring during the first 4 weeks on ART initiation [20,21,22,23,24,25,26,27,28].

Late presentation is frequently preceded by unrecognized HIV-related symptoms and prior healthcare contacts that do not result in HIV testing. Several studies have shown that PWH with AHD often present with HIV indicator conditions, such as cytopenias, lymphadenopathy, dermatological manifestations, pneumonia, or mononucleosis-like illness, representing missed opportunities for early diagnosis [29,30]. These findings highlight critical gaps in provider-initiated and indicator condition-guided HIV testing. This multicenter study aims to characterize clinical and viro-immunological features and survival among antiretroviral-naive PWH presenting with AHD in Italy, and to assess healthcare contacts and HIV-related symptoms in the year preceding diagnosis as potential missed opportunities for earlier HIV detection.

## 2. Materials and Methods

### 2.1. Study Design and Setting

This was a multicenter, retrospective, observational cohort study conducted at four HIV Italian HIV referral centers: Infectious Diseases Units of the University Hospitals of Foggia, Bari, Genoa (AOM IRCCS) and Milan (San Paolo Hospital, ASST Santi Paolo e Carlo). Infectious Diseases Unit of the University of Foggia served as the coordinating center.

### 2.2. Study Population

All antiretroviral-naive PWH diagnosed with AHD between 1 January 2019 and 31 December 2024 were eligible for inclusion. AHD was defined as a CD4 count < 200 cells/mmc and/or the presence of AIDS-defining conditions (CDC stage C) according to the Atlanta classification 1993 [31].

### 2.3. Data Collection

Demographics (age, sex, and risk factor for HIV acquisition), clinical characteristics (mode of transmission, history of AIDS diagnosis, antiretroviral regimen treatment at baseline and during follow up and opportunistic infection (OI) prophylaxis) and viro-immunological parameters (CD4+ T-cell count, HIV RNA levels) were retrieved from medical records at baseline (T0) and during annual follow up for five years (T1–T5).

Participants were proactively contacted by telephone by medical staff and interviewed regarding HIV-related symptoms and potential healthcare contacts occurred during the two years preceding their confirmed diagnosis.

In addition, outpatient medical records were reviewed to collect information on hospitalizations, specialist medical consultations and history of previous HIV screening tests. Furthermore, information on hospitalizations that occurred during the year following the HIV diagnosis were obtained from discharge summaries. All data from the participating sites were entered into a centralized multicenter database by each study site and subsequently submitted to the coordinating center for analysis.

Participants who did not complete the study were classified as those who discontinued care and were lost to follow up (LTFU). Reasons for LTFU were systematically documented and analyzed. Virological failure was defined as a confirmed HIV-RNA > 200 copies/mL within the all observation period in PWH with stable adherence to treatment (defined as on ART > 6 months).

### 2.4. Study Endpoints

The primary endpoint was the evaluation of missed opportunities for HIV diagnosis based on healthcare contacts and HIV-related symptoms in the year preceding diagnosis.

Secondary endpoints included:

- Five-year overall survival after initiation of ART among in antiretroviral-naïve PWH presenting with advanced HIV infection, compared with AIDS presenters, after initiation of ART.
- Baseline clinical and viro-immunological characterization of advanced-naïve PWH.
- Immune recovery and virological suppression at one year after ART initiation (T1).

### 2.5. Statistical Analysis

Descriptive statistics were summarized using the median and interquartile range (IQR) for quantitative variables and absolute frequencies and percentages for qualitative ones. The Shapiro–Wilk test showed that quantitative variables were not normally distributed. Associations between qualitative variables were assessed using Pearson’s chi-square test e/o Fisher’s exact test, as appropriate, while the non-parametric Wilcoxon rank-sum (Mann–Whitney U) test was used to compare quantitative variables between groups. The survival analysis was performed by applying the Kaplan–Meier estimator, with differences between survival curves evaluated by the Log-rank test.

All statistical tests were two-sided, and a *p*-value ≤ 0.05 was considered statistically significant. The analyses were conducted using STATA software, version 18 (StataCorp, College Station, TX, USA).

### 2.6. Ethical Considerations

The study was conducted in accordance with the Declaration of Helsinki, and the study was conducted using retrospectively collected and anonymized data, and therefore did not require ethical approval as determined by the Italian Drug Agency note March 20, 2008 (GU Serie Generale no. 76, 31 March 2008). Patients’ need for written informed consent was waived because of the study’s retrospective nature.

## 3. Results

Between 1 January 2019 and 31 December 2024, 658 PWH were diagnosed with HIV infection at the participating centers. Among them, 224/658 (34%) met the criteria for AHD at diagnosis and were included in the analysis, of whom 121 (54%) were AIDS presenters and 103 (46%) non-AIDS presenters. PWH were enrolled across four centers, with a significantly different distribution between groups (*p* = 0.004). Demographic and clinical data are summarized in Table 1.

The majority of PWH were male (74%), with no differences in sex distribution between individuals presenting with AIDS and those presenting without AIDS. Median age at diagnosis was 44.5 years (Inter Quartile range, IQR 36–55) and was significantly higher among AIDS presenters compared with non-AIDS presenters (50 vs. 39 years; *p* < 0.001). The most frequently reported mode of transmission was heterosexual contact (55.9%) followed by men who have sex with men (MSM, 35.6%) and people who inject drugs (PWIDs) (7%). The number of new AHD cases was evenly distributed over the study period. A higher proportion of non-Caucasian individuals was observed among PWH with AIDS at presentation compared with those without AIDS (*p* < 0.001).

At HIV diagnosis (T0), AIDS presenters showed more severe immunosuppression and higher viral replication. Median CD4 count was 31.0 cells/mm^3^ (IQR 14.0–57.0) in AIDS presenters compared with 101.0 cells/mm^3^ (IQR 42.0–156.0) in non-AIDS presenters (*p*< 0.001), with similarly lower CD4 percentages (5.2% vs. 9.1%; *p* < 0.001). Median HIV RNA levels were significantly higher in AIDS presenters (5.63 vs. 5.19 log_10_ copies/mL; *p* < 0.001). Prophylaxis for opportunistic infections at diagnosis was more frequently prescribed in AIDS presenters (86.1% vs. 65.7%; *p* < 0.001), as per management guideline.

All PWH initiated ART, most commonly bictegravir/emtricitabine/tenofovir alafenamide (BIC/FTC/TAF) (63.9%), with no significant differences in treatment distribution between the two groups. Dolutegravir (DTG)-based regimens were prescribed in 18.7%, protease inhibitor-based regimens (darunavir/cobicistat/emtricitabine/tenofovir alafenamide, DRV/c/FTC/TAF) were prescribed in 9.1%.

During follow up, both groups experienced progressive immune recovery. However, CD4 counts and percentages remained significantly lower among AIDS presenters at T1 and for CD4 percentages at subsequent time points up to T3. Differences in absolute CD4 counts were no longer statistically significant from T2 onwards. Rates of virological suppression (<50 copies/mL) were high and comparable between groups at all time points, exceeding 90% from T2 onwards. Particulalry, at T1 we observed 28% with HIV RNA between 51 and 199 cps/mL and 4.5% with HIV-RNA > 199 cps/mL. At T2, all participants suppressed to 50 cps/mL except for n. 3 participants who showed HIV RNA between 51 and 199 cps/mL.Virological failure was uncommon and did not differ significantly between groups (2.9% overall).

At last follow-up, median CD4 count was 339.0 cells/mm^3^ (182.5–520.0), with lower CD4 percentages observed among AIDS presenters (18.8% vs. 21.9%; *p* = 0.028). Available data regarding CD4 cells percentage and count over the three time-points follow up are reported in Supplementary Table S1.

Opportunistic infections during follow-up were significantly more frequent among AIDS presenters (23.3% vs. 6.0%; *p* < 0.001). Overall mortality was 6.3%, with all deaths occurring among AIDS presenters (11.6% vs. 0%; *p* < 0.001). Rates of loss to follow-up did not differ significantly between groups.

Regarding prior engagement with healthcare, AIDS presenters were less likely to report previous HIV testing compared with non-AIDS presenters (8.3% vs. 19.4%; *p* = 0.028). Notably, AIDS presenters more frequently accessed healthcare services in the year preceding HIV diagnosis (40.2% vs. 17.3%; *p* < 0.001), while no significant differences were observed in healthcare access after diagnosis.

Among 60 PWH accessing hospital care both as out- or inpatients in the year before HIV diagnosis without HIV testing, the most common clinical presentations were available for 57 and were fatigue, diarrhea and weight loss (24.5%), followed by fever with lymphadenopathy (15.7%). Persistent hematological abnormalities (pancytopenia, lymphopenia or anemia) were reported in 10.5% of cases.

Other reasons for hospital access included neoplastic diseases (Kaposi’s sarcoma, sarcomatoid carcinoma, mesenchymal neoplasm), pneumonia, oral candidiasis or esophagitis, and skin manifestations, each occurring in approximately 5% of patients. A variety of additional symptoms was reported in 24.5% of cases.

**Figure 1:**
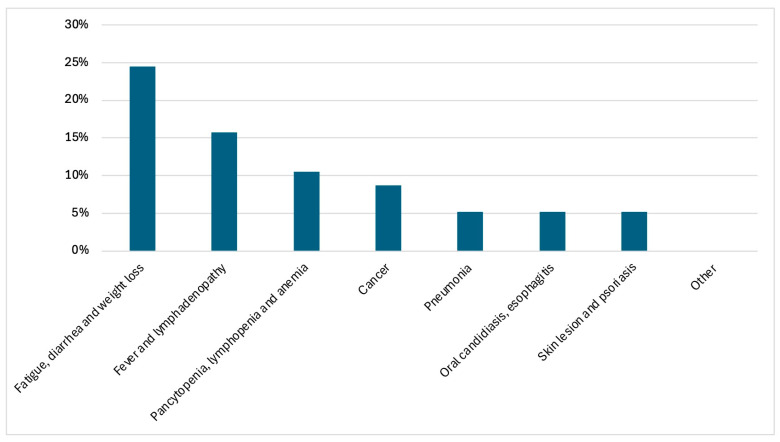
Distribution of HIV indicator conditions leading to hospital access in the year preceding HIV diagnosis (N = 57).

Overall, a wide spectrum of conditions potentially suggestive of HIV infection was observed, indicating multiple missed opportunities for HIV testing prior to diagnosis (Figure 1).

**Figure 2:**
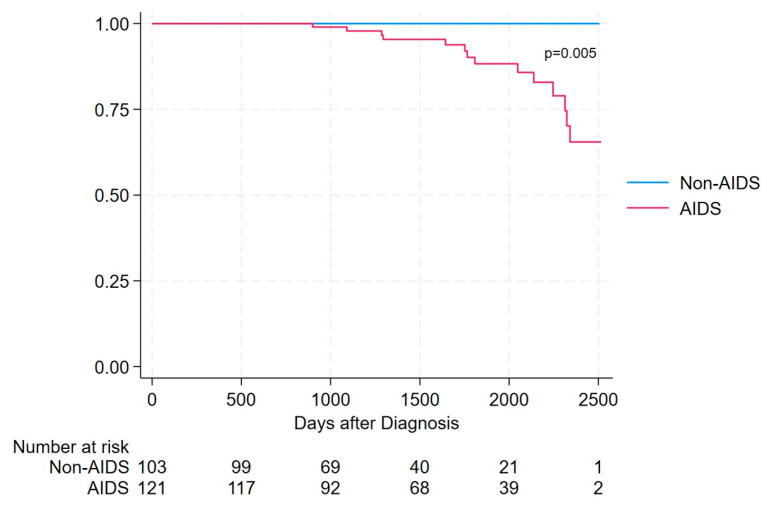
Survival in AIDS presenters vs. non-AIDS presenters, assessed using Kaplan–Meier estimator.

Overall survival differed significantly between AIDS and non-AIDS presenters. During follow-up, all deaths occurred among AIDS presenters, while no deaths were observed in the non-AIDS group. The survival after the diagnosis was significantly lower in AIDS presenters than in non-AIDS presenters (Log-rank test = 7.57, *p* = 0.005) as shown in Figure 2. Survival in the non-AIDS group remained 100% throughout the entire observation period. In contrast, AIDS presenters experienced a progressive decline in survival over time, with events occurring predominantly after the first year following HIV diagnosis. The maximum follow-up duration was approximately 7 years, corresponding to the date of database closure.

Among the 14 deceased individuals, the cause of death was ascertained in 8 cases (57.1%), while it remained unavailable in 6 cases (42.9%). Considering the overall deaths (n = 14), 5 (35.7%) were classified as AIDS-related, due to opportunistic and advanced HIV-associated conditions (including disseminated MAC with CMV esophagitis and wasting syndrome; PJP with CMV pneumonia and wasting syndrome; disseminated cryptococcosis; neurotoxoplasmosis with atypical mycobacterial infection; and HIV encephalopathy with disseminated CMV and Candida esophagitis). Three deaths (21.4%) were not AIDS-related and included squamous cell lung carcinoma, septic shock in the context of non-Hodgkin lymphoma, and acute respiratory failure with heart failure. Among cases with a known cause of death (n = 8), AIDS-related deaths accounted for 62.5% (5/8) and non-AIDS-related deaths for 37.5% (3/8).

Deceased individuals differed from survivors mainly in age and disease severity. They were older (median 55.5 vs. 43.0 years, *p* = 0.022) and all were AIDS presenters (100% vs. 51.0%, *p* < 0.001), with lower baseline CD4 counts (20 vs. 52.5 cells/mm^3^, *p* = 0.009). During follow-up, those who died showed markedly poorer immune recovery and virological control, with lower CD4 values at T1 and at the last follow-up and lower rates of viral suppression. Opportunistic infections and loss to follow-up were also significantly more frequent among deceased patients (both *p* < 0.001), whereas no significant differences were observed for sex, transmission mode, year of diagnosis, ART regimen, or access to care (Supplementary Table S2). Among AIDS presenters, most baseline characteristics were comparable between deceased individuals and survivors. No significant differences emerged in age, sex, transmission mode, baseline CD4 count, HIV RNA, ART regimen, or access to care. However, patients who died showed significantly poorer immunological recovery, with lower CD4 counts at T1 (82 vs. 236 cells/mm^3^, *p* = 0.036) and markedly lower CD4 values and percentages at last follow-up (both *p* < 0.001). They also had substantially lower rates of virological suppression at last follow-up (21.4% vs. 78.3%, *p* < 0.001) and a markedly higher burden of opportunistic infections (71.4% vs. 16.7%, *p* < 0.001). Loss to follow-up was strongly associated with mortality (85.7% vs. 22.4%, *p* < 0.001) (Suppelmentary Table S2).

## 4. Discussion

Advanced HIV disease (AHD) remains highly prevalent despite universal access to ART and widespread availability of HIV testing. In our cohort, approximately one third of newly diagnosed PWH presented with AHD, and more than half were AIDS presenters, confirming that delayed diagnosis continues to represent a major clinical and public health challenge in European settings [4,5].

These findings are consistent with recent global evidence indicating that a substantial proportion of PWH still enter care with advanced disease, even in healthcare settings where testing is readily available, suggesting persistent gaps between healthcare contact and timely HIV diagnosis [32].

Late presentation is known to be associated with increased mortality, with AIDS-related causes still contributing substantially to early deaths after diagnosis. At the same time, in the contemporary ART era, non-AIDS conditions account for an increasing proportion of overall mortality among PWH. Findings from the Italian ICONA cohort (1997–2022) show a marked decline in AIDS-related mortality over time, with non-AIDS malignancies and cardiovascular disease emerging as the leading causes of death, while individuals presenting with AIDS or severe immunosuppression continue to experience excess mortality. These observations are consistent with our results, in which deaths occurred predominantly among individuals with advanced HIV disease at presentation and included both AIDS-related conditions and non-AIDS comorbidities, reflecting the dual contribution of late diagnosis and chronic comorbidity to mortality in the modern treatment era [33].

Notably, AIDS presentation has been associated with a more than four-fold increased risk of all-cause mortality compared with other late presenters with incomplete immune recovery at two years emerging as a key mediator of long-term mortality [18]. This biological link between profound immunosuppression, impaired immune reconstitution, and excess mortality provides a plausible explanation for the poorer survival observed among AIDS presenters in our cohort.

At diagnosis, PWH exhibited low CD4 count and high levels of HIV RNA, strongly suggesting prolonged periods of undiagnosed infection, as enrollment was based on the established definition of AHD, which inherently selects for individuals with marked immunological impairment and/or high viral load.

Similar patterns have been described across European countries, where late HIV diagnosis continues to account for a substantial proportion of new diagnoses and progress toward the 2025 target of less than 20% late diagnosis remains uneven and insufficient in many settings [34]. This underscores the persistence of structural and programmatic limitations in current HIV testing strategies, which often rely on patient-initiated testing rather than systematic, provider-initiated approaches.

Although rapid initiation of modern INSTI-based regimens resulted in high rates of virological suppression and significant immune recovery within the first year, clinical outcomes remained less favorable among AIDS presenters.

Five year survival was significantly lower in this group, in line with previous European cohort studies demonstrating persistent excess mortality among individuals with AIDS, even after effective ART initiation [18,19].

In this advanced naive population, INSTI-based regimens showed high efficacy and no virological failure. BIC/FTC/TAF was the most frequently prescribed regimen and showed excellent tolerability and low discontinuation rates after 1 year, supporting its role as a preferred therapeutic option in PWH with AHD, where rapid immune recovery is critical to reduce mortality risk [33,34]. Dual therapy with dolutegravir/lamivudine (DTG/3TC) was prescribed in a minority of cases, despite emerging evidence supporting its efficacy and safety in advanced HIV infection, as demonstrated in the DOLCE study [35].

For an international readership, it may be useful to clarify that TLD (tenofovir disoproxil fumarate/lamivudine/dolutegravir) is widely recommended by WHO as a preferred first-line regimen and is commonly available as a single-tablet regimen (STR) in many low- and middle-income settings. In contrast, this specific fixed-dose STR is not routinely available in most European countries. European AIDS Clinical Society (EACS) guidelines instead recommend integrase inhibitor-based STRs that are licensed in the region, such as BIC/F/TAF, which fulfills a similar role in first-line therapy. Clarifying this distinction may help explain regional differences in regimen selection [36].

A key and novel contribution of this study is the systematic assessment of healthcare contacts and HIV-related symptoms in the period preceding diagnosis. Nearly one third of individuals had accessed healthcare services for symptoms compatible with HIV infection without undergoing HIV testing, highlighting the persistence of missed diagnostic opportunities [37]. This observation mirrors recent global findings showing a high prevalence of advanced HIV disease among individuals with prior healthcare exposure, reinforcing the notion that missed testing opportunities remain a major driver of late diagnosis [32].

The clinical presentation reported, such as cytopenia, lymphadenopathy, dermatological manifestations, and respiratory or mononucleosis-like syndromes, closely overlap with HIV indicator conditions identified in the HIDES I and II studies [29,30].

These findings underscore the insufficient integration of indicator condition-guided HIV testing into routine clinical practice, particularly in hospital-based and non-specialist settings, and reinforce its relevance as an effective strategy to promote earlier diagnosis. Evidence from European surveillance further suggests that reliance on non-systematic testing disproportionately affects vulnerable groups and contributes to sustained levels of late diagnosis across Europe [34].

Retention in care emerged as an additional critical challenge. More than one quarter of patients were lost to follow-up, with higher rates of individuals who discontinued care among AIDS presenters.

Recent ICONA data demonstrated that AHD is increasingly observed even among ART-experienced individuals, largely driven by disengagement from care, suboptimal virological control, and socio-economic vulnerability, and that incident AHD after ART initiation is associated with an increased risk of mortality [19]. Taken together, these findings suggest that late diagnosis and poor retention in care represent two interconnected and mutually drivers of persistent HIV-related morbidity and mortality, even in high-income settings. Disruptions in healthcare access during the COVID19 pandemic may have further exacerbated these challenges, particularly among vulnerable population [38,39].

Several limitations should be acknowledged. The retrospective design inherently limits the ability to infer causality and is subject to incomplete documentation and potential misclassification of clinical events. In particular, the assessment of pre-diagnosis symptoms and healthcare contacts relied partly on patient recall obtained through telephone interviews, which may be affected by recall bias and underreporting.

The high rate of loss to follow-up observed in our cohort may have influenced the estimation of virological outcomes and mortality, potentially leading to an underestimation of adverse events. In addition, detailed information on ethnicity, socioeconomic status, mental health, substance use, and structural barriers to care was not systematically available, limiting our ability to fully explore determinants of late diagnosis and retention in care.

Finally, although the multicenter design improves generalizability within Italy, the findings may not be fully applicable to settings with different healthcare systems, testing strategies, or population demographics.

Despite these limitations, this study has several notable strengths. First, it represents a large multicenter real-world cohort focusing specifically on antiretroviral-naïve PWH presenting with AHD in a European setting. The inclusion of four major Italian referral centers enhances the representativeness of the study population and improves the generalizability of the findings to similar healthcare systems.

Importantly, the integration of detailed viro-immunological, therapeutic, and clinical outcome data with a systematic evaluation of healthcare contacts before diagnosis provides a comprehensive perspective on both disease severity and missed diagnostic opportunities, an aspect rarely explored in European cohorts. 

Third, the long observational window and availability of survival data allowed for meaningful comparisons between AIDS presenters and non-AIDS advanced-naïve individuals, highlighting the persistent impact of late presentation on long-term outcomes despite access to modern antiretroviral regimens.

## 5. Conclusions

In the contemporary antiretroviral therapy era, advanced HIV disease remains a major and unresolved public health challenge in Italy.

Our findings highlight the urgent need to strengthen strategies aimed at universal opt-out screening. Systematic implementation of indicator condition-guided HIV testing into routine clinical practice, particularly in emergency departments, hospital wards, and primary care services, should be prioritized to promote earlier diagnosis, reduce the burden of advanced disease, improve retention in care, and ultimately decrease HIV-related morbidity and mortality in high-income European countries.

In addition, expanding routine opt-out HIV screening in healthcare settings could further normalize testing, increase case detection, and reduce the proportion of individuals presenting with advanced HIV disease.

## Figures and Tables

**Table 1:** Baseline characteristics, viro-immunological features, clinical outcomes, and healthcare access of antiretroviral-naïve people with advanced HIV disease, stratified by AIDS presentation.

	Total (n = 224)	Non-AIDS Presenters (n = 103)	AIDS Presenters (n = 121)	*p*-Value
Variable *				
Centers, n (%)				
Foggia	45 (20.1)	18 (17.5)	27 (22.3)	0.004
Bari	66 (29.5)	39 (37.9)	27 (22.3)	
Genoa	75 (33.5)	37 (35.9)	38 (31.4)	
Milan	38 (17.0)	9 (8.7)	29 (24.0)	
Gender, n (%)				
Male	165 (73.7)	72 (69.9)	93 (76.9)	0.413
Female	56 (25.0)	30 (29.1)	26 (21.5)	
Transgender	3 (1.3)	1 (1.0)	2 (1.7)	
Age, years, median (IQR)	44.5 (36.0–55.0)	39.0 (33.0–52.0)	50.0 (40.0–57.0)	<0.001
Mode of transmission, n (%)				
Heterosexual	124 (55.9)	56 (54.4)	68 (57.1)	0.280
MSM	79 (35.6)	37 (35.9)	42 (35.3)	
PWID	13 (5.9)	9 (8.7)	4 (3.4)	
other	6 (2.7)	1 (1.0)	5 (4.1)	
Caucasian, n (%)				
Yes	156 (69.6)	74 (71.8)	56 (46.2)	<0.001
No	68 (30.4)	29 (28.2)	65 (53.8)	
Year of diagnosis, n (%)				
2019	45 (20.1)	16 (15.5)	29 (24.0)	0.085
2020	32 (14.3)	13 (12.6)	19 (15.7)	
2021	34 (15.2%)	12 (11.7)	22 (18.2)	
2022	40 (17.9)	23 (22.3)	17 (14.0)	
2023	47 (21.0)	28 (27.2)	19 (15.7)	
2024	26 (11.6)	11 (10.7)	15 (12.4)	
CD4 count, cells/mm^3^ at T0, median (IQR)	49.5 (20.5–111.0)	101.0 (42.0–156.0)	31.0 (14.0–57.0)	<0.001
CD4% at T0, median (IQR)	7.0 (3.5–11.2)	9.1 (5.6–14.0)	5.2 (3.0–9.0)	<0.001
HIV RNA Log_10_ at T0, median (IQR)	5.45 (4.94–6.03)	5.19 (4.78–5.77)	5.63 (5.11–6.17)	<0.001
ART at T0, n (%)				
ABC/3TC/DTG	7 (3.2)	1 (1.0)	6 (5.1)	0.055
BIC/FTC/TAF	140 (63.9)	74 (72.5)	66 (56.4)	
FTC/TAF + DTG	34 (15.5)	10 (9.8)	24 (20.5)	
TAF/FTC/DRV/COBI	20 (9.1)	9 (8.8)	11 (9.4)	
Other	18 (8.3)	8 (7.9)	10 (8.6)	
OIs prophylaxis at HIV diagnosis, n (%)				
No	50 (23.4)	34 (34.3)	16 (13.9)	<0.001
Yes	164 (76.6)	65 (65.7)	99 (86.1)	
CD4 count, cells/mm^3^ at T1, median (IQR)	260.0 (184.0–360.0)	287.5 (223.5–374.0)	225.0 (165.5–340.5)	<0.014
CD4% at T1 median (IQR)	16.3 (10.8–20.6)	18.3 (14.0–24.0)	14.4 (8.6–18.6)	<0.001
HIV RNA at T1, n (%)				
<50 cps/mL	149 (84.2)	78 (85.3)	71 (82.6)	0.565
≥50 cps/mL	28 (15.8)	13 (14.3)	15 (17.4)	
Virological failure, n (%)				
No	204 (97.1)	97 (99.0)	107 (95.5)	0.218
Yes	6 (2.9)	1 (1.0)	5 (4.5)	
CD4 count, cells/mm^3^ at last FUP median (IQR)	339.0 (182.5–520.0)	359.0 (216.0–497.0)	326.0 (109.0–525.0)	0.235
CD4% last FUP median (IQR)	19.8 (13.3–26.3)	21.9 (15.2–28.2)	18.8 (11.7–25.2)	0.028
HIV RNA at last FUP, n (%)				
<50 cps/mL	162 (72.9)	86 (71.7)	76 (74.5)	0.635
≥50 cps/mL	60 (27.1)	34 (28.3)	26 (25.5)	
Active OI at last follow up, n (%)				
No	183 (84.7)	94 (94.0)	89 (76.7)	<0.001
Yes	33 (15.3)	6 (6.0)	27 (23.3)	
Death, n (%)				
No	208 (93.7)	101 (100.0)	107 (88.4)	<0.001
Yes	14 (6.3)	0 (0.0)	14 (11.6)	
Drop out, n (%)				
No	163 (72.8)	78 (75.7)	85 (70.2)	0.358
Yes	61 (27.2)	25 (24.3)	36 (29.8)	
Previous negative HIV tests, n (%)				
No	163 (86.2)	75 (80.6)	88 (91.7)	0.028
Yes	26 (13.8)	18 (19.4)	8 (8.3)	
Access to Health Care 1 year before HIV diagnosis, n (%)				
No	145 (70.7)	81 (82.7)	64 (59.8)	<0.001
Yes	60 (29.3)	17 (17.3)	43 (40.2)	
Access to HealthCare after HIV diagnosis, n (%)				
No	138 (72.6)	71 (78.0)	67 (67.7)	0.110
Yes	52 (27.4)	20 (22.0)	32 (32.3)	

*p*-values are for Pearson’s chi-square test e/o Fisher’s exact test or Wilcoxon rank-sum (Mann–Whitney U) test as appropriate; * the data refer to the available information. Abbreviations: MSM, men who have sex with men; PWID, people who inject drugs; ABC/3TC/DTG, abacavir/lamivudine/dolutegravir; BIC/F/TAF, bictegravir/emtricitabine/tenofovir alafenamide; TAF/F, tenofovir/alafenamide; DTG, dolutegravir, TAF/F/TC/DRV/c, tenfovir alfenamide/emtricitabine/darunavir/cobicistat; OIs, opportunistic infections; OI, Opportunistic Infections; FUP, Follow Up.

## Data Availability

The datasets generated during the current study are not publicly available because they contain sensitive data to be treated under data protection laws and regulations. Appropriate agreement of data sharing can be arranged after a reasonable request to the corresponding author.

## Supplementary Materials

The following supporting information can be downloaded at: https://www.mdpi.com/article/10.3390/v18030356/s1, Table S1: Descrioptive characteristics of study population stratified according AIDS-presentation; Table S2: Clinical characteristics of observed deaths; Table S3: Comparison of clinical characteristics in AIDS Presenters: deceased vs. alive
