# Supplementary material for: The Forgotten Test: Missed Opportunities for HIV Diagnosis and Survival Outcomes in Advanced HIV Disease
Source: Viruses. 2026 Mar 15;18(3):356. doi: 10.3390/v18030356 (PMC13030858; doi:10.3390/v18030356)
Supplement: Supplementary file 1 [file viruses-18-00356-s001.zip › viruses-4155099-supplementary.pdf]

Supplementary Material

Supplementary Table S1.Descrioptive characteristics of study population stratified according AIDS-presentation

	Total	***Non-AIDSpresenters***	***AIDS-presenters***	***p-value***
Variables*	***N=224***	***N=103***	***N=121***	
CD4 count, cells/mm³ at T2, median (IQR)	*335.5 (250.5-465.0)*	*357.5 (288.0-478.0)*	*298.5 (234.0-446.0)*	*0.086*
CD4 % at T2, median (IQR)	*19.9 (14.0-24.0)*	*21.1 (16.8-26.5)*	*17.9 (12.3-22.0)*	*0.002*
HIV RNA at T2, n(%)				
< 50 cps/mL	*128 (92.1)*	*69 (93.3)*	*59 (90.7)*	*0.590*
> 50 cps/mL	*11 (7.9)*	*5 (6.7)*	*6 (9.3)*	
CD4 count, cells/mm³ at T3,median (IQR)	*434.5 (303.0-534.0)*	*438.0 (358.0-520.0)*	*420.0 (288.0-534.0)*	*0.435*
CD4 % at T3, median (IQR)	*22.6 (15.0-27.4)*	*25.5 (17.0-29.7)*	*21.5 (13.9-25.3)*	*0.025*
HIV RNA at T3, n(%)				
< 50 cps/mL	*90 (94.7)*	*52 (94.5)*	*38 (95.0)*	*0.922*
≥ 50 cps/mL	*5 (5.3)*	*3 (5.5)*	*2 (5.0)*	
CD4 count, cells/mm³ at T4, median (IQR)	*421.5 (317.0-552.0)*	*422.5 (329.5-545.0)*	*418.0 (315.0-579.0)*	*0.873*
CD4 % at T4, mediuan (IQR)	*23.7 (17.5-29.0)*	*23.7 (18.9-29.3)*	*23.1 (16.9-28.8)*	*0.561*
HIV RNA at T4, n(%)				
< 50 cps/mL	*74 (94.9)*	*43 (93.5)*	*31 (96.9)*	*0.640*
≥ 50 cps/mL	*4 (5.1)*	*3 (6.5)*	*1 (3.1)*	
CD4 count, cells/mm³ at T5, median(IQR)	*465.5 (330.5-618.5)*	*465.0 (372.0-580.0)*	*482.0 (318.0-632.0)*	*0.893*
CD4 % T5, median (IQR)	*23.7 (17.8-29.0)*	*24.0 (18.1-30.0)*	*23.0 (17.2-27.0)*	*0.459*
HIV RNA at T5, n(%)				
< 50 cps/mL	*61 (93.9)*	*35 (97.2)*	*26 (89.6)*	*0.316*
≥ 50 cps/mL	*4 (6.1)*	*1 (2.8)*	*3 (10.4)*	

***Virological failure**, n(%)*				
No	*204 (97.1)*	*97 (99.0)*	*107 (95.5)*	*0.218*
Yes	*6 (2.9)*	*1 (1.0)*	*5 (4.5)*	
CD4 count, cells/mm³ at last FUP, median (IQR)	*339.0 (182.5-520.0)*	*359.0 (216.0-497.0)*	*326.0 (109.0-525.0)*	*0.235*
CD4 % last FUP, median (IQR)	*19.8 (13.3-26.3)*	*21.9 (15.2-28.2)*	*18.8 (11.7-25.2)*	*0.028*
HIV RNA at last FUP, n(%)				
< 50 cps/mL	*162 (72.9)*	*86 (71.7)*	*76 (74.5)*	*0.635*
≥ 50 cps/mL	*60 (27.1)*	*34 (28.3)*	*26 (25.5)*	

*Data are expressed as median and interquartile range (25th and 75th percentile) or n (%) as appropriate; p-values are for Pearson's chi-square test e/o Fisher's exact test or Wilcoxon rank-sum (Mann–Whitney U) test; *the data refer to the available information.*

***Abbreviations**: MSM, men who have sex with men; PWID, people who inject drugs; ABC/3TC/DTG, abacavir/lamivudine/dolutegravir;BIC/F/TAF, bictegravir/emtricitabine/ tenofovir alafenamide; TAF/F, tenofovir/alafenamide; DTG, dolutegravir, TAF/F/TC/DRV/c, tenfovir alfenamide/emtricitabine/darunavir/cobicistat; OIs, opportunistic infections*

Supplementary Table S2 – Clinical characteristics of observed deaths

	Total	***No***	***Yes***	***p-value***
****Variable***	***n=224***	***n=210***	***n=14***	
Centers, n(%)				
Foggia	*45 (20.1)*	*40 (19.0)*	*5 (35.7)*	*0.026*
Bari	*66 (29.5)*	*66 (31.4)*	*0 (0.0)*	
Genoa	*75 (33.5)*	*69 (32.9)*	*6 (42.9)*	
Milan	*38 (17.0)*	*35 (16.7)*	*3 (21.4)*	
Gender, n(%)				
Male	*165 (73.7)*	*153 (72.9)*	*12 (85.7)*	*0.610*
Female	*56 (25.0)*	*54 (25.7)*	*2 (14.3)*	
Transgender	*3 (1.3)*	*3 (1.4)*	*0 (0.0)*	
Age, years, median (IQR)	*44.5 (36.0-55.0)*	*43.0 (36.0-55.0)*	*55.5 (44.0-59.0)*	*0.022*
Mode of transmission, n(%)				
Heterosexual	*124 (55.9)*	*116 (55.8)*	*8 (57.1)*	*0.495*
MSM	*79 (35.6)*	*74 (35.6)*	*5 (35.7)*	
PWID	*13 (5.9)*	*13 (6.2)*	*0 (0.0)*	
Other	*6 (2.8)*	*9 (2.4)*	*1 (7.1)*	
Year of diagnosis, n(%)				
2019	*45 (20.1)*	*41 (19.5)*	*4 (28.6)*	*0.415*
2020	*32 (14.3)*	*29 (13.8)*	*3 (21.4)*	

2021	34 (15.2)	31 (14.8)	3 (21.4)	
2022	40 (17.9)	37 (17.6)	3 (21.4)	
2023	47 (21.0)	46 (21.9)	1 (7.1)	
2024	26 (11.6)	26 (12.4)	0 (0.0)	
**AIDS, n(%)**				
AIDS-presenters	121 (54.0)	107 (51.0)	14 (100.0)	<0.001
Non-AIDS-presenters	103 (46.0)	103 (49.0)	0 (0.0)	
**CD4 count, cells/mm^3 at T0, median (IQR)**	49.5 (20.5-111.0)	52.5 (25.0-117.0)	20.0 (10.0-50.0)	0.009
**CD4 % at T0, median (IQR)**	7.0 (3.5-11.2)	7.0 (3.6-11.2)	6.0 (2.4-11.2)	0.693
**HIV RNA Log10 at T0, median (IQR)**	5.45 (4.94-6.02)	5.42 (4.93-6.00)	5.87 (5.20-6.25)	0.066
**cART at T0, n(%)**				
ABC/3TC/DTG	7 (3.2)	5 (2.4)	2 (16.7)	0.091
BIC/FTC/TAF	140 (63.9)	133 (64.3)	7 (58.3)	
FTC/TAF + DTG	34 (15.5)	33 (15.9)	1 (8.3)	
TAF/FTC/DRV/COBI	20 (9.1)	18 (8.7)	2 (16.7)	
Other	18 (8.2)	18 (8.7)	0 (0.0)	
**OIs prophylaxis at HIV diagnosis, n(%)**				
No	50 (23.4)	47 (23.3)	3 (25.0)	0.562
Yes	164 (76.6)	155 (76.7)	9 (75.0)	
**CD4 count, cells/mm3 at T1, median (IQR)**	260.0 (184.0-360.0)	262.0 (185.0-364.0)	82.0 (65.0-201.0)	0.018
**CD4 % at T1, median (IQR)**	16.3 (10.8-20.6)	16.3 (10.9-21.0)	3.0 (2.9-14.4)	0.021
**HIV RNA at T1, n(%)**				
<50 cps/mL	149 (84.2)	146 (84.9)	3 (60.0)	0.178
≥50 cps/mL	28 (15.8)	26 (15.1)	2 (40.0)	
**Virological failure, n(%)**				
No	204 (97.1)	193 (96.9)	11 (100)	0.721
Yes	6 (2.9)	6 (3.1)	1 (0)	
**CD4 count, cells/mm^3 at last FUP, median (IQR)**	339.0 (182.5-520.0)	356.5 (202.0-531.0)	33.5 (14.0-76.0)	<0.001
**CD4 % last FUP, median (IQR)**	19.8 (13.3-26.3)	21.1 (14.0-26.7)	6.0 (3.0-16.9)	<0.001
**HIV RNA at last FUP, n(%)**				
<50 cps/mL	162 (73.0)	159 (76.4)	3 (21.4)	<0.001
≥50 cps/mL	60 (27.0)	49 (23.6)	11 (78.6)	
**OI at last follow up, n(%)**				
No	183 (84.7)	179 (88.6)	4 (28.6)	<0.001
Yes	33 (15.3)	23 (11.4)	10 (71.4)	
**Drop out, n(%)**				
No	163 (72.8)	161 (76.7)	2 (14.3)	<0.001
Yes	61 (27.2)	49 (23.3)	12 (85.7)	
**Previous HIV tests, n(%)**				
No	163 (86.2)	155 (85.6)	8 (100.0)	0.602
Yes	26 (13.8)	26 (14.4)	0 (0.0)	
**Access to Health Care 1 year before HIV diagnosis, n(%)**				
No	145 (70.7)	138 (70.8)	7 (70.0)	0.958
Yes	60 (29.3)	57 (29.2)	3 (30.0)	
**Access to Health Care after HIV diagnosis, n(%)**				
No	138 (72.6)	129 (72.9)	9 (69.2)	0.754

Yes	52 (27.4)	48 (27.1)	4 (30.8)	

Supplementary Table S3 - Comparison of clinical characteristics in AIDS Presenters: deceased vs alive

	Total	AIDS - presenters -vivi	Morti	p-value
**Variable**	**n=121**	**n=107**	**n=14**	
Centers, n(%)				
Foggia	27 (22.3)	22 (20.6)	5 (35.7)	0.103
Bari	27 (22.3)	27 (25.2)	0 (0.0)	
Genoa	38 (31.4)	32 (29.9)	6 (42.9)	
Milan	29 (24.0)	26 (24.3)	3 (21.4)	
**Gender, n(%)**				
Male	93 (76.9)	81 (75.7)	12 (85.7)	0.791
Female	26 (21.5)	24 (22.4)	2 (14.3)	
Transgender	2 (1.7)	2 (1.9)	0 (0.0)	
**Age, years, median (IQR)**	50.0 (40.0-57.0)	50.0 (39.0-56.0)	55.5 (44.0-59.0)	0.123
**Mode of transmission, n(%)**				
Heterosexual	68 (57.1)	60 (57.1)	8 (57.1)	0.764
MSM	42 (35.3)	37 (35.2)	5 (35.7)	
PWID	4 (3.4)	4 (3.8)	0 (0.0)	
Other	5 (4.2)	4 (3.9)	1 (7.1)	
**Year of diagnosis, n(%)**				
2019	29 (24.0)	25 (23.4)	4 (28.6)	0.571
2020	19 (15.7)	16 (15.0)	3 (21.4)	
2021	22 (18.2)	19 (17.8)	3 (21.4)	
2022	17 (14.0)	14 (13.1)	3 (21.4)	
2023	19 (15.7)	18 (16.8)	1 (7.1)	
2024	15 (12.4)	15 (14.0)	0 (0.0)	
**CD4 count, cells/mm^3 at T0, median (IQR)**	31.0 (14.0-57.0)	31.0 (15.0-57.0)	20.0 (10.0-50.0)	0.251
**CD4 % at T0, median (IQR)**	5.2 (3.0-9.0)	5.0 (3.0-8.4)	6.0 (2.4-11.2)	0.580
**HIV RNA Log10 at T0, median (IQR)**	5.6 (5.1-6.2)	5.6 (5.1-6.1)	5.9 (5.2-6.3)	0.341
cART at T0, n(%)				
ABC/3TC/DTG	6 (5.1)	4 (3.8)	2 (16.7)	0.159
BIC/FTC/TAF	66 (56.4)	59 (56.2)	7 (58.3)	
FTC/TAF + DTG	24 (20.5)	23 (21.9)	1 (8.7)	
TAF/FTC/DRV/COBI	11 (9.4)	9 (8.6)	2 (16.7)	
Other	10 (8.5)	10 (9.5)	0 (0.0)	
**OIs prophylaxis at HIV diagnosis, n(%)**				
No	16 (13.9)	13 (12.6)	3 (25.0)	0.370
Yes	99 (86.1)	90 (87.4)	9 (75.0)	
**CD4 count, cells/mm3 at T1, median (IQR)**	225.0 (165.5-340.5)	236.0 (168.0-345.0)	82.0 (65.0-201.0)	0.036
**CD4 % at T1, median (IQR)**	14.4 (8.6-18.6)	14.5 (9.1-18.9)	3.0 (2.9-14.4)	0.057
**HIV RNA at T1, n(%)**				
<50 cps/mL	78 (85.7)	75 (87.2)	3 (60.0)	0.147
≥50 cps/mL	13 (14.3)	11 (12.8)	2 (40.0)	
**Virological failure, n(%)**				
No	107 (95.5)	96 (95.0)	11 (100.0)	0.450

Yes	*5 (4.5)*	*5 (5.0)*	*0 (0.0)*	
CD4 count, cells/mm^3 at last FUP, median (IQR)	*326.0 (109.0-525.0)*	*347.0 (193.0-544.0)*	*33.5 (14.0-76.0)*	*<0.001*
CD4 % last FUP, median (IQR)	*18.8 (11.7-25.2)*	*19.0 (13.6-25.8)*	*6.0 (3.0-16.9)*	*<0.001*
HIV RNA at last FUP, n(%)				
<50 cps/mL	*86 (71.7)*	*83 (78.3)*	*3 (21.4)*	*<0.001*
≥50 cps/mL	*34 (28.3)*	*23 (21.7)*	*11 (78.6)*	
OI at last follow up, n(%)				
No	*89 (76.7)*	*85 (83.3)*	*4 (28.6)*	*<0.001*
Yes	*27 (23.3)*	*17 (16.7)*	*10 (71.4)*	
Drop out, n(%)				
No	*85 (70.2)*	*83 (77.6)*	*2 (14.3)*	*<0.001*
Yes	*36 (29.8)*	*24 (22.4)*	*12 (85.7)*	
Previous HIV tests, n(%)				
No	*88 (91.7)*	*80 (90.9)*	*8 (100.0)*	*0.485*
Yes	*8 (8.3)*	*8 (9.1)*	*0 (0.0)*	
Access to Health Care 1 year before HIV diagnosis, n(%)				
No	*64 (59.8)*	*57 (58.8)*	*7 (70.0)*	*0.737*
Yes	*43 (40.2)*	*40 (41.2)*	*3 (30.0)*	
Access to Health Care after HIV diagnosis, n(%)				
No	*67 (67.7)*	*58 (67.4)*	*9 (69.2)*	*0.586*
Yes	*32 (32.3)*	*28 (32.6)*	*4 (30.8)*	
